# Molecular pathways underlying sympathetic autonomic overshooting leading to fear and traumatic memories: looking for alternative therapeutic options for post-traumatic stress disorder

**DOI:** 10.3389/fnmol.2023.1332348

**Published:** 2024-01-08

**Authors:** Márcia Azevedo, Raquel Martinho, Ana Oliveira, Paulo Correia-de-Sá, Mónica Moreira-Rodrigues

**Affiliations:** ^1^Laboratory of General Physiology, Department of Immuno-Physiology and Pharmacology and Center for Drug Discovery and Innovative Medicines (MedInUP), School of Medicine and Biomedical Sciences (ICBAS), University of Porto (UP), Porto, Portugal; ^2^Laboratory of Pharmacology and Neurobiology, Department of Immuno-Physiology and Pharmacology and Center for Drug Discovery and Innovative Medicines (MedInUP), School of Medicine and Biomedical Sciences (ICBAS), University of Porto (UP), Porto, Portugal

**Keywords:** physiology of fear, contextual fear memory, pathophysiology of fear, traumatic contextual memory, catecholamines, adrenoceptors

## Abstract

The sympathoadrenal medullary system and the hypothalamic-pituitary-adrenal axis are both activated upon stressful events. The release of catecholamines, such as dopamine, norepinephrine (NE), and epinephrine (EPI), from sympathetic autonomic nerves participate in the adaptive responses to acute stress. Most theories suggest that activation of peripheral β-adrenoceptors (β-ARs) mediates catecholamines-induced memory enhancement. These include direct activation of β-ARs in the vagus nerve, as well as indirect responses to catecholamine-induced glucose changes in the brain. Excessive sympathetic activity is deeply associated with memories experienced during strong emotional stressful conditions, with catecholamines playing relevant roles in fear and traumatic memories consolidation. Recent findings suggest that EPI is implicated in fear and traumatic contextual memories associated with post-traumatic stress disorder (PTSD) by increasing hippocampal gene transcription (e.g., *Nr4a*) downstream to cAMP response-element protein activation (CREB). Herein, we reviewed the literature focusing on the molecular mechanisms underlying the pathophysiology of memories associated with fear and traumatic experiences to pave new avenues for the treatment of stress and anxiety conditions, such as PTSD.

## 1 Introduction

Homeostasis comprises a set of coordinated physiological processes to sustain the steady state of an organism. Regulation of involuntary physiologic processes by the autonomic nervous system is a typical example of such processes (Walter, 1934). The “freeze, fight or flight” response, often referred to as the acute stress reaction, is an animal’s reaction to threats. This response is believed to prepare the organism to become capable of dealing with a stressor (Bracha, 2004; Lipov, 2014). The sympathoadrenal medullary system and the hypothalamic-pituitary-adrenocortical system have emerged as crucial pathways to stress responses in mammals (Selye, 1946; McEwen et al., 1974; Schommer et al., 2003). Consolidation of emotional memories is enhanced by neurotransmitters generated and released in response to stress (Matthews et al., 2012; McLaughlin et al., 2015). While these systems are adaptive in the context of acute stressful events, ongoing stress can produce chronic overactivation of the sympathoadrenal medullary system and of the hypothalamic-pituitary-adrenocortical axis. This prolonged activation is associated with the development and progression of anxiety, depression, and/or post-traumatic stress disorder (PTSD) (VanItallie, 2002).

The risk of suicide death is twice as high for people with PTSD compared to those without the condition (Fox et al., 2021). When people have symptoms of additional conditions like anxiety and depression, this association is stronger (Bantjes et al., 2016). Therefore, investigation of the variables involved in the resolution of stressful responses is deeply needed (Buckner et al., 2017; Brådvik, 2018; Twenge et al., 2018). This review provides an overview of the literature about the molecular mechanisms underlying fear memory physiology and traumatic memory pathophysiology. The involvement of catecholamines, namely norepinephrine (NE) and epinephrine (EPI), and adrenoceptors (ARs) in the development of stress and anxiety disorders will be detailed here, given that our hypothesis is that a thorough understanding of this interplay may pave the way for novel therapies to address stress and anxiety diseases, such as PTSD.

## 2 Hypothalamus-pituitary-adrenocortical system and the sympathoadrenal medullary system

**FIGURE 1:**
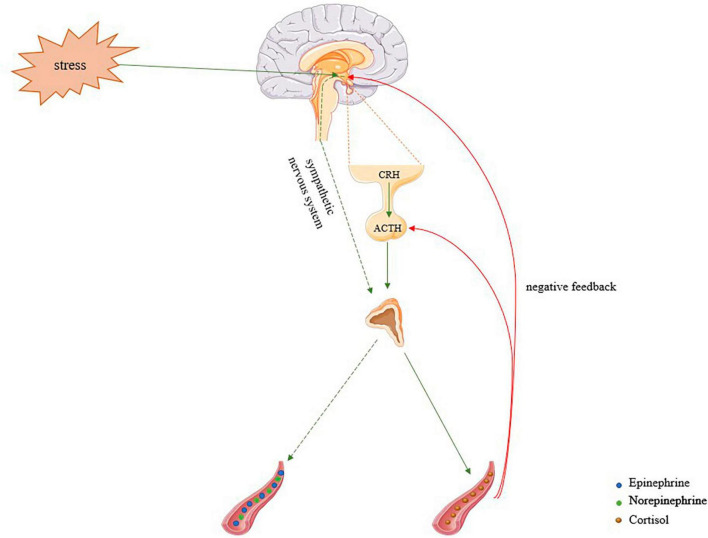
Activation of the hypothalamic-pituitary-adrenal system and the sympathoadrenal medullary system. Stress activates the limbic system in the midbrain. The amygdala stimulates the hypothalamus to release the corticotropin-releasing factor (CRF), thus inducing the production of adrenocorticotropic hormone (ACTH) by the pituitary gland. ACTH is released into the bloodstream and can act on the adrenal gland, triggering the cortical area of the gland to produce corticosteroids (Smith and Vale, 2006; Tank and Lee Wong, 2015). On the other hand, the hypothalamus also activates the Sympathetic Nervous System and through autonomic nerves triggers the release of norepinephrine and epinephrine from the adrenal medulla into the bloodstream (Smith and Vale, 2006; Tank and Lee Wong, 2015).

The hypothalamic-pituitary-adrenocortical system and the sympathoadrenal medullary system are both activated during a stressful scenario and work together to regulate the “freeze, fight, or flight” response (Bracha, 2004) by releasing stress hormones (corticosteroids and catecholamines) into the bloodstream (Figure 1; Timio et al., 1979; Floriou-Servou et al., 2021). According to preclinical research, hypothalamic-pituitary-adrenocortical and sympathoadrenal medullary systems cooperate to regulate the consolidation and retrieval of fear memories (Eiden, 2013; Hauer et al., 2014).

Upon a stressful event, the limbic system in the midbrain becomes active. The hypothalamus is activated by various systems, including immune, hormonal, and neural systems. Amygdala stimulation is a possible way of activating the hypothalamus and, consequently, two pathways may concur (Figure 1; Matheson et al., 1971; Beaulieu et al., 1988). The amygdala stimulates the hypothalamus and the anterior hypothalamus releases corticotropin-releasing factor (CRF), inducing the production of adrenocorticotropic hormone (ACTH) by the pituitary gland (Antoni et al., 1983; Gibbs et al., 1983). ACTH is released into the bloodstream and acts on the adrenal gland triggering the cortical region of the gland to produce corticosteroids, which then allows the organism to boost its metabolism in response to freeze, fight, or flight responses (Selye, 1956; Seaward, 2011). The hypothalamus also participates in intermediate and prolonged responses to aversive events by triggering the release of NE and EPI from the adrenal medulla into the bloodstream, therefore regulating the physiologic response to stressors (Smith and Vale, 2006; Tank and Lee Wong, 2015). The immune system is another mechanism of activation of the hypothalamus, through cytokines, such as IL-10, resulting in altered levels of ACTH and glucocorticoids (Petrowski et al., 2018). Therefore, the hypothalamus is a crucial part of the stress system, collaborating with other important brain regions and peripheral tissues and organs to mobilize an effective adaptive response against stressors (Li et al., 2021).

As mentioned, catecholamines, in particular EPI and NE, are two of the most important stress hormones (Romero and Butler, 2007), which together with glucocorticoids, are the main mediators of “freeze, fight or flight” responses (Bracha, 2004). Synergy between β_2_-ARs and glucocorticoid receptors has been reported. For instance, the β_2_-AR signaling pathway regulates the glucocorticoid receptor nuclear translocation and increases their affinity to steroids, as shown by the *in vivo* interaction of corticosteroids and inhaled long-acting β_2_-agonists on nuclear translocation of glucocorticoid receptors in human airway cells using immunocytochemistry (Usmani et al., 2005). This is also true in the opposite direction, since corticosteroids upregulate β_ 2_-AR transcription and regulate both their number and binding to adenylate cyclase (Yates et al., 1996; Roth et al., 2002). Furthermore, adrenal glucocorticoids directly stimulate phenylethanolamine-*N*-methyltransferase (Pnmt), the enzyme that catalyzes the conversion of NE to EPI in the adrenal medulla among other tissues of the body. Sharara-Chami et al. (2010) demonstrated that high dosages of an exogenous corticosteroid increase Pnmt and catecholamine synthesis in the absence of stress when adrenocorticotropic hormone is low, probably independently of adrenal corticosterone concentration.

Adrenomedullary responses are extremely rapid because the sympathetic nervous system (SNS) directly innervates the adrenal medulla. Adrenomedullary responses can occur before the onset of actual stress, due to the involvement of several central nervous system (CNS) regions, specifically the hippocampus (Cattane et al., 2022). NE is released from postganglionic sympathetic neurons: the second neurons in the sympathetic pathway that are part of the autonomic nervous system. Through this release, the adrenomedullary output (80% EPI and 20% NE) is followed in a coordinated manner by SNS (White and Porterfield, 2012).

## 3 Theories about the role of peripheral catecholamines in memory formation and consolidation

Catecholamines are hydrophilic and do not cross the blood-brain barrier (BBB) which prevents it from acting directly in the CNS (Weil-Malherbe et al., 1959). Catecholamines may indirectly act on brain areas responsible for learning and memory. Mounting evidence suggests the effects of stress hormones and, consequently peripheral and central β-AR activation, in memory consolidation and reconsolidation (Akirav and Maroun, 2013). The resulting effects of β-blockage with propranolol suggest a role of catecholamines in the consolidation of emotional memories (Cahill et al., 1994). This and other studies (van Stegeren et al., 1998) indicate that β-ARs activation influences long-term declarative memory consolidation for stressful emotional events triggering the release of adrenergic hormones.

**FIGURE 2:**
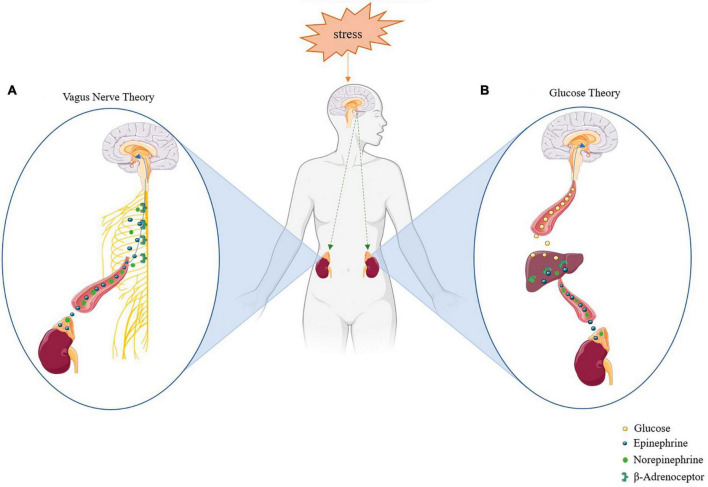
The vagus nerve and glucose as mediators of catecholamine effects in strengthening emotional memories. Stress activates the limbic system, and amygdala stimulates the hypothalamus which activates the Sympathetic Nervous System and thus triggers catecholamines release in the adrenal gland and may follow one or both of the following pathways. **(A)** Catecholamines may activate the β-adrenoceptors present in peripheral vagus nerve afferents (Schreurs et al., 1986; Gordan et al., 2015). The nucleus tractus solitarius receives the afferent vagal input (Sumal et al., 1983) and its neurons may release norepinephrine (NE) onto the locus coeruleus, which in turn may release NE in the basolateral amygdala and hippocampus (Clark et al., 1999; Miyashita and Williams, 2004, 2006; Chen and Williams, 2012). On the other hand, catecholamines released in the adrenal gland **(B)** may activate the hepatic β-adrenoceptors, causing a release of glucose into the bloodstream (John et al., 1990). Blood glucose, through glucose transporters, can cross the blood-brain barrier and potentiate the synthesis of neuromodulators that will activate signaling pathways in the basolateral amygdala and hippocampus (McNay and Gold, 2002). Both paths activate signaling mechanisms that may affect the process of consolidation of fear and traumatic memory formation.

Most theories on how catecholamines strengthen emotional memory have been focusing on the activation of peripheral β-ARs. One such hypothesis implicates the activation of β-ARs present in the vagus nerve (Miyashita and Williams, 2006; Figure 2A). As a matter of fact, EPI intraperitoneal injections increase vagal nerve firing, an effect that may be blocked by β-AR antagonists, such as sotalol or propranolol (Sternberg et al., 1985; Miyashita and Williams, 2006). The peripheral administration of β_2_-AR antagonist, ICI 118,551, induces amnesia in passive avoidance memory (Davies and Payne, 1989), as well as impairment of the contextual fear memory (Oliveira et al., 2018). In addition, ICI 118,551 along with the β_1_-AR antagonist, betaxolol, reduced the facilitation of field excitatory synaptic potentials induced by AR agonists in the basolateral amygdala (Abraham et al., 2008). Likewise, increases in endogenous amounts of NE in the basolateral amygdala improved memory formation after emotionally arousing experiences (Chen and Williams, 2012; Rudy, 2020).

Another theory considers that increased glucose release by the liver could be a mediator of catecholamine effects in the brain (Gold, 2014; Figure 2B). According to this theory, glucose may potentiate the synthesis of neuroactive mediators participating in the process of memory formation and/or consolidation (Durkin et al., 1992). Behavioral tests performed in food-deprived rats show that these animals have difficulties in memory retention contrary to the rats that had normal access to food exhibiting high glucose reserves (Talley et al., 2000).

## 4 Effect of epinephrine in fear contextual memory

The effect of catecholamines was initially evaluated in adrenal medullectomy studies (Kvetnansky et al., 1979). Adrenal medullectomy involves surgically removing the medullar zone of the adrenal gland. Putative damage of the cortex and modification of corticosteroid, NE, chromogranin A, and neuropeptide Y release are limitations of this technique (Harrison and Seaton, 1966). By using this approach, it is also left unclear whether the reported effects are caused by EPI alone or by EPI plus NE combined. Therefore, we should investigate whether EPI and NE play different roles regarding the influence of catecholamines on emotional memory. This can be achieved using inhibitors of the Pnmt enzyme, thus preventing EPI formation from NE (Bondinell et al., 1983). Notwithstanding this, usage of Pnmt inhibitors still has disadvantages due to inhibition of α-ARs (Feder et al., 1989) and monoamine oxidase (Mefford et al., 1981), resulting in the disruption of normal physiological responses. This can be overcome by decreasing the expression of the enzyme. The EPI-deficient mouse model developed by Ebert et al. (2004) and Bao et al. (2007) is unable to convert NE to EPI since Pnmt is not expressed. These EPI-deficient mice are viable, fertile, and have no gross developmental impairments (Ebert et al., 2004), thus offering greater advantages over other methodological approaches to investigate the physiological influence of EPI among other catecholamines (Ebert et al., 2004; Bao et al., 2007; Sun et al., 2008).

Fear conditioning is a behavioral paradigm by which animals acquire abilities to predict aversive events by associating the stimulus to a specific context or tone (Curzon et al., 2009). Toth et al. (2013) were the first to use mice lacking Pnmt to evaluate EPI deficiency in contextual fear memory. Toth et al. (2013) and Alves et al. (2016) concluded that EPI-deficient mice exhibited reduced contextual memory after fear conditioning compared with wild-type mice, thus suggesting that contextual fear memory requires EPI synthesis. Data from these experimental studies also showed that EPI deficiency affects both fear memory retrieval and consolidation, but was devoid of effect on fear memory acquisition (Toth et al., 2013; Alves et al., 2016). Even though EPI strengthens fear contextual memory when the animals are in an aversive context, no differences were observed when the context was changed but the same auditory cue was present. Indeed, it is believed that hippocampus is not implicated in auditory fear conditioning memory, but it is involved in contextual fear conditioning (Rudy et al., 2004). Although auditory and contextual fear memories involve some common brain regions, such as amygdala (Goosens and Maren, 2001), these findings suggest that strengthening of contextual fear memory by EPI is hippocampal-dependent (Toth et al., 2013; Alves et al., 2016).

Deficits in contextual fear memory observed in EPI-deficient mice were restored by treating the animals with EPI, isoprenaline (a non-selective β-AR agonist), or fenoterol (a selective β_2_-AR agonist) (Alves et al., 2016). Interestingly, EPI strengthening of fear contextual memory was long-lasting, as it was still observed 1 month after the training of control mice compared to EPI-deficient mice (Oliveira et al., 2018). This effect seems to be mediated by peripheral β_2_-AR activation considering that intraperitoneal administration of sotalol (non-selective β-AR antagonist) and ICI 118,551 (selective β_2_-AR antagonist) reversed the EPI memory strengthening effect (Alves et al., 2016; Oliveira et al., 2018). Neither NE nor the selective β_1_-AR agonist, dobutamine, restored the contextual fear memory deficits observed in EPI-deficient mice (Alves et al., 2016).

On the other hand, the glucose effect in improving memory was characterized by an inverted U-dose response becoming detrimental above a given hyperglycemic threshold in humans [50 g, PO (Parsons and Gold, 1992)], mice [5 g/kg, PO (Messier and Destrade, 1988)], and rats [500 mg/kg, SC injection (Gold, 1986)], which is similar to EPI. The rise in glucose levels in the bloodstream during a stressful event may foster the production/release of certain neuromodulators affecting memory formation and consolidation in the inhibitory passive avoidance task (Sandusky et al., 2013). However, passive avoidance task does not allow for an accurate investigation of fear associative memory, contrary to fear conditioning (Ögren and Stiedl, 2010).

Interestingly, wild-type mice experienced higher range of glycemic variations following fear conditioning in comparison to that observed in EPI-deficient mice, which might presumably be caused by EPI release (Alves et al., 2016; Oliveira et al., 2018). The fact that glucose contributes to glycogen storage in astrocytes might explain its action as a mediator of contextual fear memory strengthening. Increased glycogen stores may contribute to sustain glucose supply to the brain and facilitate the synthesis of pyruvate or lactate (Newman et al., 2011). Pyruvate, lactate, and glucose may provide the necessary energy for processes triggered during fear memory formation (Brown et al., 2004). Glucose availability may also directly affect the membrane potential of glucose-sensing neurons in the hippocampus (de Araujo, 2014). In a more recent study, contextual fear memory in EPI-deficient mice was enhanced after glucose (30 mg/kg) administration, suggesting that moderate blood glucose levels during contextual fear memory acquisition and retrieval are necessary. Along with these findings, simultaneous administration of sub-effective doses of EPI (0.01 mg/kg) and glucose (10 mg/kg) enhanced contextual fear memory in EPI-deficient mice, whereas separate administration of these sub-effective doses was insufficient to enhance contextual fear memory, suggesting that Ad and glucose may act in synergy to strengthen contextual fear memory. These findings may reinforce the theory that glucose may be an important part of the peripheral to central pathway of contextual fear memory strengthening by EPI (Oliveira et al., 2023a). In this sense, peripheral EPI and subsequent glucose supply to the brain may promote hippocampal-dependent contextual fear memory (Alves et al., 2016; Oliveira et al., 2018, 2023a). In addition, it was observed enhanced contextual fear memory after insulin treatment, even in adrenaline absence, which may indicate a key role of insulin in contextual fear memory, possibly by increasing local cerebral energy use (Oliveira et al., 2023b). Insulin may facilitate glucose entry in neurons and astrocytes through insulin receptors activation and promotion of Glut-4 translocation to the hippocampus cell membrane (Pearson-Leary and McNay, 2016). In addition, in insulin administration group there was an increase in plasma catecholamines and a possible increase in the uptake of dopamine to hippocampus cells (Oliveira et al., 2023b). The influence of glucose and insulin in fear memory modulation may be important in diabetes. In fact, insulin plays a role in the regulation of the oxidative state and neuronal apoptosis in the CNS. These mechanisms might be responsible for the association between insulin activity changes and neuronal degeneration in the diabetic brain. In this context, it was observed resistance to fear extinction, increased fear generalization, along with increases in anxiety-like behaviors in a type 1 diabetes animal model (Lin et al., 2018; de Souza et al., 2019).

## 5 Molecular mechanisms underlying epinephrine effect in contextual fear memory

Long-term memory (LTM) consolidation and persistence involves synaptic remodeling as a consequence of long-lasting changes in gene expression (Josselyn et al., 2001; Dudai, 2002) and protein synthesis (Schafe and LeDoux, 2000; Kim et al., 2022). Glucose consumption increases contextual fear memory and results from a hippocampal-dependent associative learning mechanisms (Glenn et al., 2014), which may be mediated by phosphorylation (activation) of the cAMP response-element protein (CREB) (Impey et al., 1998). Because CREB is a transcription factor linked to LTM formation in various systems (Alberini, 2009), studying CREB-mediated signaling pathways in the hippocampus may be useful to understand the mechanisms underlying fear memory formation. The role of CREB in fear memory consolidation has been demonstrated in genetically altered mice (Kida et al., 2002). Phosphorylated CREB (pCREB) promotes the synthesis of immediate-early genes, like the nuclear receptor 4A (NR4A) transcription factor family (Darragh et al., 2005). The CREB interaction domain of the histone acetyltransferase CREB-binding protein with pCREB is needed for *Nr4a* gene expression after learning (Bridi et al., 2017). Learning-induced *Nr4a* expression is upregulated by histone deacetylase (HDAC) inhibition, which seems crucial for LTM improvement (Bridi and Abel, 2013).

The *Nr4a* gene family seems to be important for contextual fear memory formation and consolidation (Malkani and Rosen, 2000; von Hertzen and Giese, 2005; Hawk et al., 2012). The three members of *Nr4a* transcription factors gene family, namely *Nr4a1* (NGF-B/Nur77), *Nr4a2* (NURR1), and *Nr4a3* (NOR1), belong to the immediate-early genes category (Milbrandt, 1988; Law et al., 1992; Ohkura et al., 1994). Interestingly, the deficits in contextual fear memory observed in EPI-deficient mice occur along with decreases in *Nr4a1*, *Nr4a2*, and *Nr4a3* mRNA gene transcripts in the hippocampus compared to the levels observed in wild-type mice. At the mRNA level, the *Nr4a2* gene transcription increased significantly in the hippocampus after administration of EPI to EPI-deficient mice (Oliveira et al., 2018). In addition, glucose administration in EPI-deficient mice increased hippocampus Nr4a3 gene transcription after contextual fear conditioning (Oliveira et al., 2023a). These findings strengthen our conclusion that the EPI-glucose pathway fosters the transcription of immediate-early genes in the hippocampus to promote contextual fear memory consolidation (Oliveira et al., 2018, 2023a).

Expression of Nr4a immediate early genes increases after a few hours of the stimulus and may influence other target genes such as brain-derived neurotrophic factor (*Bdnf*) gene. The BDNF peptide is also mainly generated in areas associated with learning and memory, as is the case of the hippocampal formation (Dugich-Djordjevic et al., 1995; Kawamoto et al., 1996). Overexpression of this neurotrophin participates in contextual fear conditioning (Hall et al., 2000; Mizuno et al., 2012). Hippocampal mRNA expression of *Bdnf* was increased in glucose-treated or insulin-treated EPI-deficient mice compared to vehicle-treated mice, thus suggesting that up-regulation of this gene associated with exogenous glucose or insulin results in increased contextual fear memory (Oliveira et al., 2023a,b). This situation may be due to insulin’s action on cerebral energy expenditure since it is known that insulin promotes glucose transporter Glut-4 translocation to the plasma membrane of hippocampus cells which increases glucose availability inside the cell (Pearson-Leary and McNay, 2016; Oliveira et al., 2023b).

## 6 Effect of catecholamines in traumatic contextual memory focusing on patients and animal models

Emotions can affect memory under certain circumstances, such as during acute or prolonged extreme stressful conditions (LaBar, 2007). Indeed, stress responses are crucial for preserving homeostasis when threatening situations occur (Cannon, 1939; McEwen, 1998). Nevertheless, serious anxiety and stress disorders can emerge whenever stress responses are inadequately triggered or become dysregulated. Severe dreadful experiences, such as death threats, violent crimes, warfare experience, sexual assault, especially if causing acute or chronic pain may predispose some individuals to psychiatric stress-related disorders, such as PTSD (North et al., 2012). In addition, adverse experiences may affect the maturation of the brain (van der Bij et al., 2020). PTSD affects nearly 6.8% of adults in the USA, with a significant women vs. men predisposition (Gradus, 2007). In fact, women are more predisposed to symptoms of several psychological diseases including stress, anxiety, depression, and PTSD (Gao et al., 2020; Xiong et al., 2020). The risk rate of lifetime PTSD could rise to 30% in populations that are highly exposed to stress, namely during armed conflicts and natural disasters (Breslau et al., 1998; Grinage, 2003).

Post-traumatic stress disorder patients suffer from three main conditions leading to specific symptomatology: re-experiencing, avoidance, and hyperarousal (North et al., 2012). Patients with PTSD constantly avoid any social environment or circumstance that may trigger memories of the event. Moreover, the hyperarousal state affects the ability to concentrate and sleep and causes a heightened startle response. Furthermore, hyperactivation of the SNS is a characteristic of PTSD patients (Shalev et al., 1992; Sherin and Nemeroff, 2011). In fact, increased stress hormones in plasma and urine, namely NE and EPI, have been observed in PTSD patients (Yehuda et al., 1992, 1998; Lemieux and Coe, 1995; Delahanty et al., 2005); this was also observed in a systematic meta-analysis (Pan et al., 2018).

Notwithstanding, the aforementioned considerations, PTSD is not a homogeneous disorder. The dissociative PTSD subtype has been associated with clinical severity, early life trauma and comorbid psychiatric disorders. This subtype exhibits the opposite pattern of the conventional amygdala hyperactivity with low prefrontal cortex (PFC) activation which is defined by low amygdala activation with a hyperactive prefrontal area (Lanius et al., 2010). In dissociative PTSD, the relationship between hyperactive prefrontal areas and reduced amygdala activation is complicated and requires further elucidation. The hypoactivity of the amygdala may contribute to emotional detachment and to reduce the capacity to understand and integrate emotional experiences in these patients (Forster et al., 2017). A negative correlation between EPI and NE levels and symptoms of peritraumatic dissociation exists (Delahanty et al., 2003). This suggests that highly dissociative individuals may not react physiologically to the initial traumatic event, thus dissociating and distancing themselves from trauma. On the other hand, activation of the prefrontal cortex may function as a compensatory mechanism. Individuals afflicted by dissociative PTSD may undertake excessive cognitive control processes as a coping mechanism for intense emotions or traumatic experiences. The PFC, particularly the medial PFC, has a role in executive tasks, such as emotional regulation and cognitive control (Waugh et al., 2014; Alexandra Kredlow et al., 2022). Therefore, a dissociative state may occasionally be exacerbated by increased PFC activity as a protective mechanism to suppress or control trauma. This polarity between hyper- and hypo-arousal may be just one of many facets of emotion-regulation mechanisms. However, there are many exceptions to what is considered the general rule and the clinical reality scenarios may be even more complex. Certain patients have unpredictable and unstable responses, meaning that their subjective emotions and physiological parameters do not correspond with each other (Kozlowska, 2007; González et al., 2017). Together, these findings suggest that the unique symptomatology of PTSD might be derived from hyper- and hypo-arousal of the amygdala and the PFC, which may explain the putative differences in catecholamines levels in these patients.

The simultaneous occurrence of PTSD with various mental disorders, such as depression and anxiety, is relatively common. This gives rise to comorbid PTSD conditions associated to a wide anxiety/depression spectrum (Koenig et al., 2018; Malgaroli et al., 2018), which may be correlated to disturbances in the functioning of hypothalamic-pituitary-adrenal axis (Pan et al., 2018).

To overcome environmental and inter-individual variability, animal models of PTSD have been instrumental to understand the neurobiological aspects of PTSD in terms of individual susceptibility, clinical response to stress, and prediction of therapeutic outcomes. Previous reviews have corroborated several animal models for stress paradigms, including physical stress, predator stress, and social defeat stress (Verbitsky et al., 2020). Since the traumatic experience necessary for the onset of PTSD can be considered an unconditioned stimulus associated with a conditioned stimulus (the context), variations of contextual fear conditioning are described as PTSD animal models (VanElzakker et al., 2014). One of these models uses multiple electric shocks as the unconditioned stimulus to simulate the unpleasant traumatic event that might trigger PTSD (Verma et al., 2016). This model combines prolonged exposure time with high-intensity currents, to produce long-lasting symptoms. Animals normally exhibit increased respiratory rate and freezing, as well as anxiety-like behaviors in response to foot shocks. This is also consistent with the pathological nature of PTSD and its symptoms (Li et al., 2006; Zhang et al., 2012). Moreover, animals exposed to repeated foot shocks may acquire phobia as a result of the traumatic experience (Delahanty et al., 2005), which fits the phobia occurrence in PTSD patients (Orsillo et al., 1996). The fear contextual memory that arises from the training days might be a representation of trauma reminders in PTSD patients (Li et al., 2006). The electric shock model may be explored to develop in-context treatments since repeated exposure to the environments and cues associated with stressors is the clinical counterpart of exposure therapies (Verbitsky et al., 2020). Thus, a physical stressor, such as electric foot shocks, might be a valuable resource to access the full range of signs (and symptoms) presented in this disorder.

Using the latter animal model, Martinho et al. (2020) investigated the role of catecholamines in PTSD (Moreira-Rodrigues and Grubisha, 2022; Abumaria et al., 2023); data indicate that mice with PTSD display higher contextual traumatic memory, along with increased levels of NE and EPI in adrenal glands and higher levels of EPI in the plasma compared to controls. The persistence of contextual traumatic memories led to an anxiety-like behavior and resistance to traumatic memory extinction with a possible involvement of hippocampal *Nr4a2* and *Nr4a3* genes (Martinho et al., 2020; Moreira-Rodrigues and Grubisha, 2022; Abumaria et al., 2023).

## 7 Putative therapeutic approaches to traumatic contextual memory formation and reconsolidation in PTSD

Selective serotonin reuptake inhibitors (SSRIs), like paroxetine and sertraline, are the only drugs approved by FDA and EMA for treating PTSD in human patients (Davidson et al., 2001; McRae and Brady, 2001; Friedman et al., 2007). SSRIs have only marginal effects on the severity and progression of PTSD symptoms compared to a control group (Bowers and Ressler, 2015; Hoskins et al., 2015). A meta-analysis of 55 studies found a 29% average dropout rate, thus indicating that most individuals do not tolerate or respond to existing PTSD treatments, including SSRIs (Lee et al., 2016). The fact that the majority of these drugs either fail to reach their maximal effect or cause severe adverse side effects agrees with the view that there is still an unmet clinical need in the treatment of PTSD. There is a potential suitability of molecules involved in inflammatory, immune, and hypothalamic-pituitary-adrenal axis responses to diagnose and treat PTSD. One studied molecule was matrix metalloproteinase 9 (MMP9), an extracellular matrix-degrading enzyme (Chevalier et al., 2021).

In the catecholamine biosynthesis pathway, dopamine-β-hydroxylase (DBH) is responsible for the conversion of DA to NE. Mice lacking DBH exhibit diminished contextual fear memory, which is restored by the administration of isoprenaline (a non-selective β-AR agonist) (Murchison et al., 2004). Nepicastat, a highly active reversible DBH inhibitor, effectively reduces NE in peripheral and central tissues both in rats (Bonifacio et al., 2015; Loureiro et al., 2015) and dogs (Stanley et al., 1997). By effectively modulating SNS hyperactivation, nepicastat may be a useful strategy to treat PTSD. As a matter of fact, Martinho et al. (2021) demonstrated that nepicastat significantly decreased DBH activity in the adrenal glands, which led to a gradual decrease in NE and EPI over 24 h (Moreira-Rodrigues and Grubisha, 2022; Abumaria et al., 2023). Nepicastat-treated PTSD mice showed reduced traumatic contextual memory and reduced anxiety-like behavior compared to control litter mates (Martinho et al., 2021; Moreira-Rodrigues and Grubisha, 2022; Abumaria et al., 2023).

Concerning the molecular pathways involved in the formation and consolidation of contextual fear and traumatic memories, besides *Nr4a* gene products, NPAS4 may also have a role. NPAS4 encodes for the activity-dependent transcription factor known as neuronal PAS domain protein 4 expressed in the CA3 hippocampal region and may play a role in neuronal regulation (Yun et al., 2010; Ploski et al., 2011). In fact, *Npas4* has been associated with fear memory and contextual fear conditioning (Ramamoorthi et al., 2011). According to some, the amount of hippocampal NPAS4 mRNA gene transcripts is positively correlated with hippocampal activation (Drouet et al., 2018) and is implicated in the control of a transcriptional program that includes the *Bdnf* gene (Hall et al., 2000; Lin et al., 2008; Mizuno et al., 2012). Interestingly, nepicastat increased *Npas4* and *Bdnf* genes transcription in the hippocampus, thus strengthening the hypothesis that these gene products play a crucial role in the weakening of traumatic contextual memories by replacing these with neutral contextual memories (Martinho et al., 2021; Moreira-Rodrigues and Grubisha, 2022; Abumaria et al., 2023). Since nepicastat application was made after the traumatic event but before contextual tests, catecholamines may strengthen traumatic memory at first, but afterward, this drug decreases catecholamines and, thus, the contextual memory may become neutral upon repetitive application of the DBH inhibitor before contextual exposition days. This approach mimics the combination of pharmacotherapy (DBH inhibitor) with psychotherapy (context exposition in a safe environment) for the treatment of PTSD, which may contribute to resilience and coping with the trauma.

Since no DBH inhibitor has received marketing approval due to poor DBH selectivity, low potency, and/or significant side effects, DBH gene silencing with small interference RNA technology may be a potential new therapeutic alternative for PTSD, particularly in patients with increased sympathetic activity. There are already several FDA-approved agents for metabolic diseases that are small interfering RNA (siRNA) based therapies and exert their effects by RNA interference (RNAi) of their target mRNA (Padda et al., 2023).

Furthermore, the reduction of the aforementioned sympathetic autonomic overshooting in PTSD patients may be achieved through inhibition of β-ARs activity (Murchison et al., 2004; Ramos et al., 2005). In fact, administration of propranolol (a peripheral and central β-AR antagonist) actually prevents the onset and progression of PTSD symptoms in human subjects exposed to traumatic situations, in particular when administered prior to trauma memory reactivation (Pitman et al., 2002; Brunet et al., 2008; Young and Butcher, 2020). Systemic propranolol disrupts the consolidation and reconsolidation of traumatic memories, but these putative beneficial effects are undermined due to unwanted side effects (e.g., gastrointestinal disturbances, bradycardia, fatigue, and sleep disorders) (Shukla and McGowan, 2009), along with memory deficits (e.g., decreased memory consolidation in non-aversive tasks and impairments in memory reconsolidation in least aversive tasks) (Villain et al., 2016).

Considering these facts, administration of the peripheral β-AR antagonist, sotalol, could overcome the unwanted central effects of propranolol. In fact, PTSD animals treated with sotalol exhibited decreased traumatic contextual memories, anxiety-like behaviors, and plasma catecholamines compared to vehicle-treated animals (Martinho et al., 2022). These effects of sotalol most probably result from a reduction of catecholamine effects in peripheral β-ARs leading to attenuation of the sympathetic autonomic overactivity, which indirectly down-modulates traumatic contextual memories (Martinho et al., 2022). With the data available so far, one can only speculate about the involvement of the sympathetic-induced vagal nerve drive and/or glucose release theories (Clark et al., 1999; Miyashita and Williams, 2004, 2006; Chen and Williams, 2012; Gold, 2014) to explain the results showing decreases in *Nr4a1* mRNA transcripts in the hippocampus of sotalol-treated mice (Martinho et al., 2022). Our hypothesis is that sotalol may be repurposed as a possible alternative to SSIRs for PTSD treatment, especially in patients with peripheral sympathetic hyperactivity. Sotalol is currently used in ventricular and supra-ventricular arrhythmias, despite the associated risk of unwanted pro-arrhythmic effects due to prolongation of the QT interval (Somberg et al., 2010). Upon increasing the dosage of sotalol it abruptly interrupts the response to adrenergic stimuli by competitively blocking β-ARs. While the sotalol dosage to treat PTSD may be lower, the reversible inhibition of DBH may be a better option for this disease condition due to slower installation yet a more sustained decrease of adrenergic responses.

These data show that hippocampus genes may play a role in the modulation of traumatic contextual memories in PTSD animals: *Nr4a* family genes in sotalol treatment (Martinho et al., 2022), and *Npas4* and *Bdnf* genes in nepicastat treatment (Martinho et al., 2021; Moreira-Rodrigues and Grubisha, 2022; Abumaria et al., 2023). This difference may also be related to different treatment regimens. Nepicastat treatment may have affected formation, expression, retrieval, consolidation or reconsolidation processes of contextual traumatic memory, whereas treatment with sotalol does not affect memory formation since no treatment with sotalol was applied in the traumatic event days, contrary to that occurring with nepicastat.

Furthermore, data indicate that EPI may trigger persistence of traumatic contextual memories in PTSD, possibly by increasing the transcription of *Nr4a2* and *Nr4a3* genes in the hippocampus (Martinho et al., 2020; Moreira-Rodrigues and Grubisha, 2022; Abumaria et al., 2023). Therefore, therapeutic options aiming at specifically blocking EPI effects could decrease unwanted side effects compared to other broader effect options, as could be the case for PNMT gene silencing in PTSD.

## 8 Conclusion

Catecholamines play a role in the consolidation of fear memories, which are crucial for developing an adaptive defensive mechanism and responding to adverse environmental stressors (Romero and Butler, 2007). Catecholamines are released upon activation of the sympathoadrenal medullary system after stressful events, thus initiating the important homeostasis response of “freeze, fight or flight” (Timio et al., 1979; Bracha, 2004). When fear responses are inaccurately triggered and/or regulated, homeostasis is compromised and traumatic memories can emerge leading to pathological conditions, such as anxiety and PTSD. Some, but not all, PTSD patients exhibit sympathetic autonomic overshooting (Shalev et al., 1992; Sherin and Nemeroff, 2011) with increased plasma and urinary levels of catecholamines (Yehuda et al., 1992; Lemieux and Coe, 1995; Delahanty et al., 2005).

Through peripheral β_2_-ARs activation of vagal nerve inputs to the CNS and/or by triggering glucose release from the liver, EPI may play a crucial role in strengthening contextual fear and traumatic memories (Miyashita and Williams, 2006; Chen and Williams, 2012; Toth et al., 2013; Gold, 2014; Alves et al., 2016; Oliveira et al., 2018, 2023a,b; Martinho et al., 2022; Moreira-Rodrigues and Grubisha, 2022; Abumaria et al., 2023). Subsequently, the transcription of immediate-early genes of the *Nr4a* family results in specific protein synthesis in the hippocampus to allow contextual fear and traumatic memories formation and consolidation (Oliveira et al., 2018, 2023a,b; Martinho et al., 2020, 2022). In this sense, our hypothesis is that sotalol (a peripheral non-selective β-ARs blocker) (Martinho et al., 2022) and nepicastat (a DBH inhibitor) (Martinho et al., 2021; Moreira-Rodrigues and Grubisha, 2022; Abumaria et al., 2023) might help to decrease PTSD occurrence by weakening abnormal traumatic memories formation and consolidation, and may support in the formation of neutral contextual memories. From the molecular point of view, we show that hippocampus genes may play a role in the modulation of traumatic contextual memories in PTSD animals: *Nr4a* family genes in sotalol treatment (Martinho et al., 2022), and *Npas4* and *Bdnf* genes in nepicastat treatment (Martinho et al., 2021; Moreira-Rodrigues and Grubisha, 2022; Abumaria et al., 2023). Thus, the take-home message of this review is that PTSD patients with sympathetic autonomic overshooting may benefit from downregulating dysregulated catecholamine effects and DBH or PNMT gene silencing may be a therapeutic option. An approach that mimics the combination of pharmacotherapy with psychotherapy may contribute to resilience and coping with the trauma.

## Author contributions

MA: Writing – original draft, Writing – review and editing. RM: Writing – original draft. AO: Writing – original draft. PC-d-S: Funding acquisition, Resources, Writing – review and editing. MM-R: Conceptualization, Funding acquisition, Resources, Supervision, Writing – original draft, Writing – review and editing.
